# Effect of High Nighttime Temperatures on Growth, Yield, and Quality of Two Wheat Cultivars During the Whole Growth Period

**DOI:** 10.3390/plants13213071

**Published:** 2024-10-31

**Authors:** Danping Li, Yanjun Xiao, Lei Guo, Baoxue Shan, Xiukun Liu, Xiaoyan Duan, Ata-ur Rehman, Can Guo, Wenjia Zhang, Haosheng Li, Jianjun Liu, Xin Gao, Xinyou Cao

**Affiliations:** 1 Crop Research Institute, Shandong Academy of Agricultural Sciences/National Engineering Research Center of Wheat and Maize/State Key Laboratory of Wheat Improvement/Key Laboratory of Wheat Biology and Genetic Improvement in North Yellow & Huai River Valley/Shandong Provincial Technology Innovation Center for Wheat, Jinan 250100, China; 13121259599@163.com (D.L.); xiaoyanjun0@163.com (Y.X.); leiguo@nwafu.edu.cn (L.G.); shanbaoxue1217@163.com (B.S.); liuxk0224@163.com (X.L.); duanxiaoyan0329@163.com (X.D.); guocan97@126.com (C.G.); ycwenjia@163.com (W.Z.); lihaosheng810@163.com (H.L.); 2 Shandong Academy of Agricultural Sciences, Jinan 250100, China; 3 Gulbali Institute, Charles Sturt University, Wagga Wagga, NSW 2650, Australia; arehman@csu.edu.au

**Keywords:** climate warming, high nighttime temperatures, winter wheat, yield, quality

## Abstract

It is a consensus that Earth’s climate has been warming. The impact of global warming is asymmetric, that is, there is more substantial warming in the daily minimum surface air temperature and lower warming in the maximum surface air temperature. Previous studies have reported diurnal temperature differences greatly affecting winter wheat yield. However, only a few studies have investigated the impact of global warming on the growth and yield of winter wheat, yet the influence of night warming on quality has not been deeply evaluated. In this study, two wheat cultivars were used as materials: Jimai 44 (JM44) with strong gluten and Jimai 22 (JM22) with medium gluten, to explore the effects of high nighttime temperatures (HNTs) on the growth, yield, and quality of wheat. The results show that HNTs significantly shortened seedling emergence and anthesis periods in both cultivars compared with ambient temperatures (ATs). In addition, HNTs increased the respiration rate at anthesis and grain-filling stages, impeding wheat pollination and grain maturity. HNTs also accelerated leaf senescence and increased the number of sterile spikelets and plant height, but decreased the effective tiller number, the number of spikes per unit area, and grains per spike. As a result, the grain yield of JM22 and JM44 was decreased by 24.6% and 21.2%, respectively. Moreover, HNTs negatively influenced the flour quality of the two wheat cultivars. The current findings provide new insights into the effects of HNTs on the growth, development, yield, and quality of different wheat genotypes during the whole growth period.

## 1. Introduction

Globally, the average surface temperature of 2021 was 1.44 ± 0.20 °C above the pre-industrial baseline (1850–1900) [1]. According to the State of the Global Climate 2021 (WMO-No, 1290), the long-term trend is clear even though global temperatures are not rising yearly [2]. The effects of high temperatures on crops are adverse [3]. Due to its geographical and environmental adaptability, winter wheat (*Triticum aestivum* L.) is more sensitive to global warming than other cereals [4]. The average wheat grain yield (GY) has been reported to decrease by 8.5% to 28.5% when the temperature increase ranged between 1 °C and 3 °C [5]. Maintaining wheat GY in the future therefore poses a serious challenge to breeders and farmers if the current trend in warming continues.

Several studies have shown that daily minimum temperatures rise much faster than daily maximum temperatures [6,7]. Compared with diurnal thermal peaks, HNTs may affect crops’ further growth and developmental stages and therefore affect the yields of crop plants over larger spatial areas and for more extended periods [8]. Investigation into crop phenology changes has important implications for understanding the response and adaptation of crops to climate change [9]. Previous studies have recorded the length of each growth period and consistently found that HNTs shortened the growth period of wheat, especially the duration of pre-anthesis [10,11,12]. However, the effects of the shortened growth period on wheat biomass and GY were inconsistent. Therefore, the effect of HNTs needs to be further evaluated by more detailed characteristics, such as leaf age, tiller number, leaf area index (LAI), plant height, and biomass accumulation at each developmental stage.

With the increased human population, about 200 million tons of additional wheat grain will be required by 2050 [13]. The impact of HNTs on crop yield is important, and has been addressed [5,14,15]. However, the reported effects of HNTs on wheat GY were not consistent in the previous studies. Negative impacts have been reported between wheat GY and HNTs under controlled environments [16], field conditions [11,17], and modeling studies [18]. The average GY of different wheat genotypes under HNT stress was reduced by 20.3%, with the highest reduction of 41.4% and the lowest reduction of 6.9% [19]. On the contrary, increased temperature positively affected GY in some studies [14,15,20,21]. This inconsistency may be attributed to the differences in cultivar, treatment, year, location, and other factors. Given that different cultivars respond to high temperatures differently [14], systematically studying the individual performances of cultivars may reveal the causes of conflicting reports on the warming impacts. The three yield components, including the number of spikes (NS) per unit area, grain number per spike (GNS), and thousand-kernel weight (TKW), can be affected by phenological changes and the accumulation and distribution of assimilates [22,23]. There are different agronomic traits associated with yield components for various cultivars. Therefore, it is important to clarify the relationship between yield components and the agronomic traits to reveal the responses of wheat to HNTs.

Wheat is consumed by humans in various forms, such as bread, steamed buns, noodles, and biscuits [24]. With the improvement of life quality, the nutritional and processing quality of wheat are gaining growing importance, and the requirements for improving wheat flour quality have been discussed [25,26]. While wheat quality is governed innately by genetics, the quality traits are also subjected to the impacts of growing conditions such as the agronomic treatments, soil, and climatic parameters, which determine the final quality of the grain [27,28,29]. Increased temperatures in winter and spring can alter wheat’s morphology, growth, and physiological characteristics and thus affect its yield and quality. Although the impacts of climate warming on crop phenology, biomass, and GY have been investigated extensively, few studies reported the effects on grain quality, which has become a significant concern [26,30]. The commercial value of wheat is determined by the processing quality of flour [31], a combination of many defined parameters that determine the quality of wheat. Multiple phenotypic traits of cereals, flour, dough, and final products are worth efforts to determine the overall quality and optimal end-use products [32]. However, studies on the effect of HNTs on wheat processing quality throughout the growth period have not been conducted extensively.

Given that gluten characteristics can well reflect the processing quality [33], determining the gluten content and index can provide further explanation for the changes in wheat quality parameters. Moreover, due to differences in flour components, whiteness can also be affected as an important appearance quality. Flour pasting characteristics are essential quality parameters and are significantly related to the quality scores of breads or noodles [34], but the effect of HNTs on flour pasting is rarely reported. Dough rheological properties are among the most critical indicators for studying wheat flour quality. Previous studies have indicated that water absorption (WA) and dough development time (DDT), as well as stability time (ST) affect the processing quality of the final wheat flour products, especially baking quality [35,36]. The tensile test evaluated the quality of processing ability, which was positively correlated with food product volume [37]. Further field trials involving different cultivars in different environments are required to determine the effects of genotypes and climate warming and their interaction [38].

This study treated a strong-gluten wheat cultivar and a medium-gluten wheat cultivar with HNTs throughout the growth period. The leaf age, tiller number, LAI, plant height, and dry matter accumulation were measured at each wheat growth and development stage. The relative chlorophyll content (SPAD value), net photosynthetic rate during the day, and nighttime respiration rate of flag leaves were determined at the grain-filling stage. Yield components and economic coefficients were examined at the maturity stage. Quality traits, particularly the properties of gluten and starch of wheat grain and flour, were investigated to elucidate the effects of HNTs on the reproductive growth period, yield, and quality of winter wheat.

## 2. Results

### 2.1. Impacts of HNT–Genotype Interactions on Wheat Growth and Development, Leaf Area, Biomass Accumulation, and Distribution

A comparison of ATs with HNTs showed an increase from 1.3 °C to 1.8 °C, with an average increase of 1.8 °C in December, 1.3 °C in January, 1.4 °C in February, 1.6 °C in March, and 1.8 °C in April and May (Figure S1).

**Figure 1:**
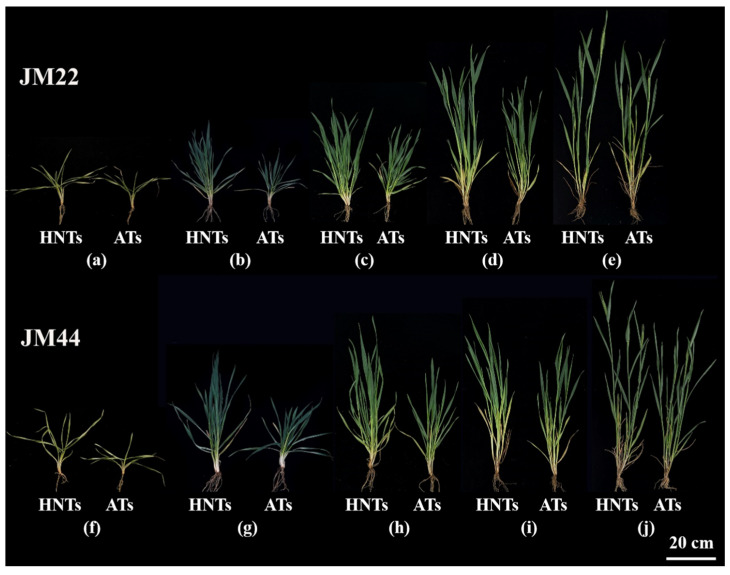
Differences between high nighttime temperatures (HNTs) and ambient temperatures (ATs) of Jimai22 (JM22) and Jimai44 (JM44) at overwintering (**a**,**f**), re-greening (**b**,**g**), jointing (**c**,**h**), booting (**d**,**i**), and heading (**e**,**j**) stages, with the scale bar of 20 cm.

The leaf age differed significantly between the two wheat cultivars under the same conditions, with JM44 displaying more mature leaves than JM22. HNTs accelerated wheat growth; the leaf age at HNTs was significantly more advanced than at ATs (Figure 1). At the beginning of the overwintering period, wheat cultivars JM22 and JM44 developed more rapidly at HNTs than at ATs (Figure 1). Hence, their leaves, matched by age and position, are more advanced in development at HNTs. These results indicate that the increased temperatures promote plant growth and development with a significant effect on leaf age (Figure 1 and Figure S2).

**Figure 2:**
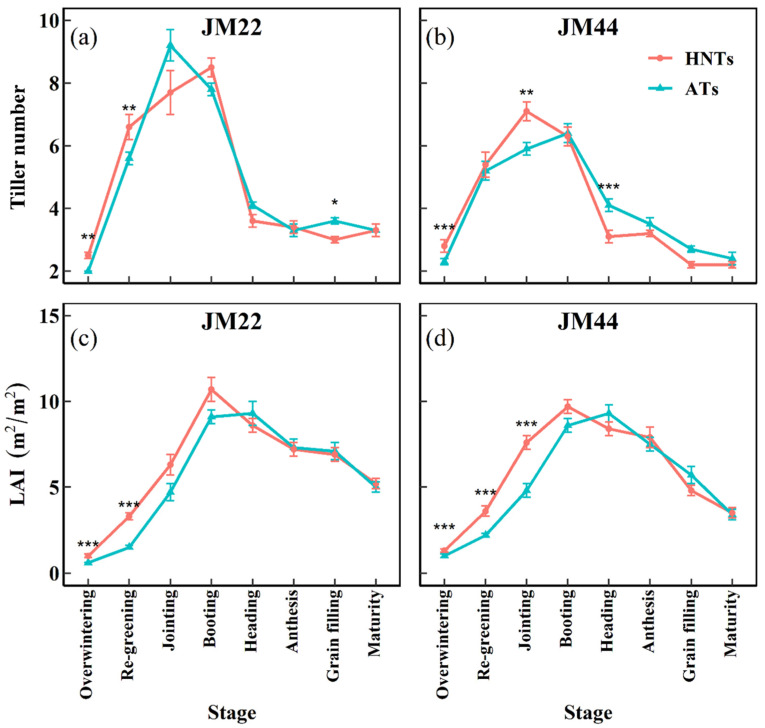
Tiller number (**a**,**b**) and leaf area index (LAI) (**c**,**d**) of Jimai22 (JM22) and Jimai44 (JM44) with days after sowing under the high nighttime temperatures (HNTs) and ambient temperatures (ATs). Analysis of variance (ANOVA) and the least significant difference (LSD) were used to test the significance of differences. *, **, and *** indicate a significant difference between HNTs and ATs at the 0.05, 0.01, and 0.001 levels, respectively.

Moreover, the two wheat cultivars showed a consistent pattern of tiller development under HNT treatments and ATs. From overwintering to maturity, the tiller number first increased and then decreased, reaching the peak at the jointing or booting stage (Figure 2a,b). For JM22, the HNT treatment significantly increased the tiller number at overwintering and re-greening stages, while it decreased the tiller number at the grain-filling stage (Figure 2a). Similarly, for JM44, the HNT treatment significantly increased the tiller number at overwintering and jointing stages, while it decreased the tiller number at the heading stage (Figure 2b).

The two wheat cultivars showed a consistent pattern in the LAI under the HNT treatment and ATs. From overwintering to maturity, the LAI first increased and then decreased, and the LAI at the booting stage was the largest (Figure 2c,d). For JM22, the HNT treatment significantly increased the LAI at overwintering and re-greening stages yet had no significant effect on the LAI from the jointing to maturity stage (Figure 2c). Similarly, for JM44, the HNT treatment significantly increased the LAI from the overwintering to jointing stage yet had no significant effect on the LAI from the booting to maturity stage (Figure 2d). This indicates that the HNT treatment promoted leaf development at an early stage.

**Figure 3:**
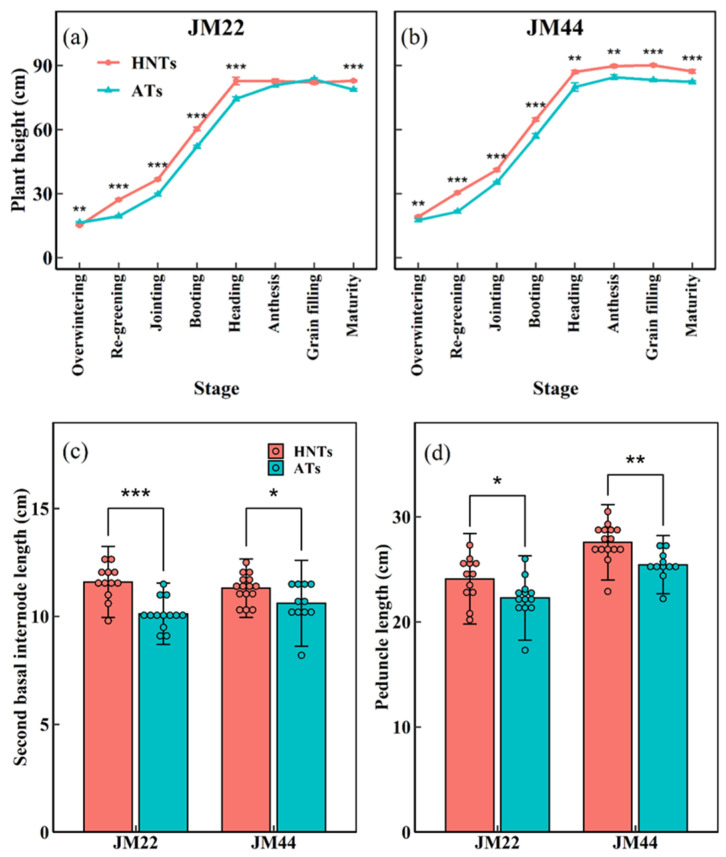
Plant height (**a**,**b**), the length of second basal internode (**c**) and peduncle (**d**) of Jimai22 (JM22) and Jimai44 (JM44) with days after sowing under high nighttime temperatures (HNTs) and ambient temperatures (ATs). Analysis of variance (ANOVA) and the least significant difference (LSD) were used to test the significance of differences. *, **, and *** indicate a significant difference between HNTs and ATs at the 0.05, 0.01, and 0.001 levels, respectively.

The plant height of JM22 was increased at most stages by increased nighttime temperatures, except for the overwintering, anthesis, and grain-filling stages (Figure 3a). However, JM44 showed an increased plant height at all stages at HNTs (Figure 3b). Additionally, at the re-greening stage, the difference in plant height of JM22 and JM44 between HNTs and ATs reached its maximum, and the plant height of JM22 and JM44 was 28.3% and 29.2% higher at HNTs than at ATs, respectively. The measurement data of the mature main stem indicates that HNTs mainly increased the growth of the second basal internode and peduncle, resulting in a higher plant height (Figure 3c,d). Patterns of changes in the plant height of the two wheat cultivars at HNTs and ATs were almost consistent.

**Figure 4:**
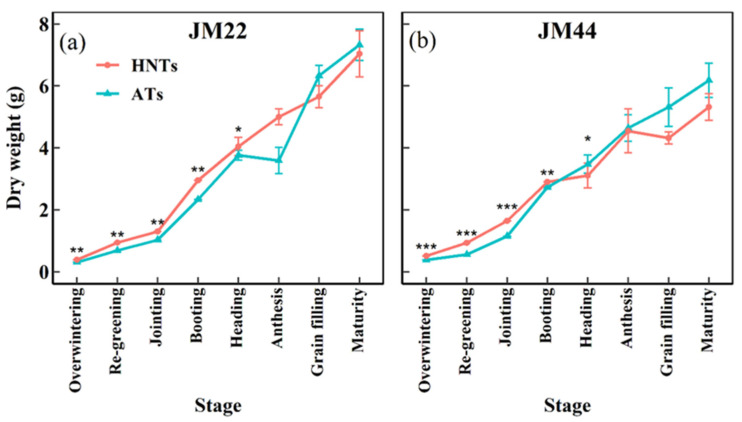
Dry weight per plant of Jimai22 (JM22) (**a**) and Jimai44 (JM44) (**b**) at maturity stage under high nighttime temperatures (HNTs) and ambient temperatures (ATs). Analysis of variance (ANOVA) and the least significant difference (LSD) were used to test the significance of differences. *, **, and *** indicate a significant difference between HNTs and ATs at the 0.05, 0.01, and 0.001 levels, respectively.

**Figure 5:**
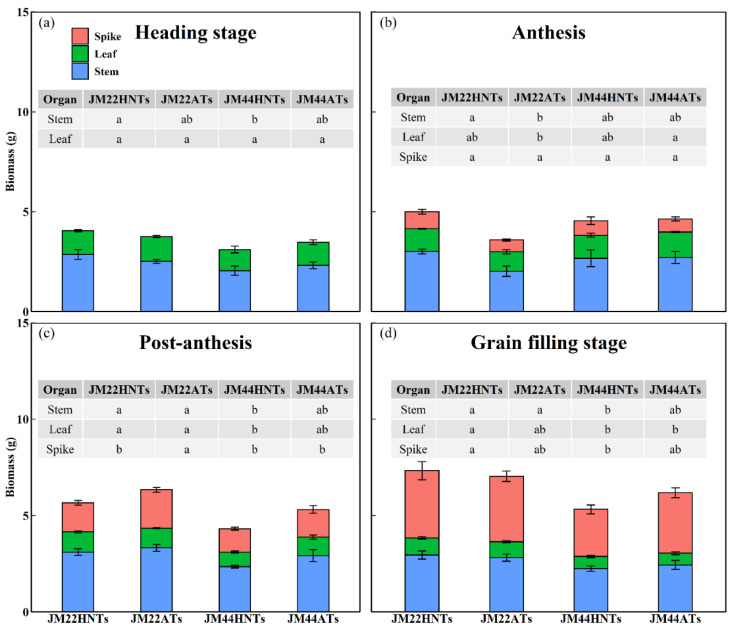
Biomass allocation of stem, leaf and spike at heading (**a**), anthesis (**b**), post-anthesis (**c**), and grain-filling stages (**d**) of Jimai22 (JM22) and Jimai44 (JM44) under high nighttime temperatures (HNTs) and ambient temperatures (ATs). Analysis of variance (ANOVA) and the least significant difference (LSD) were used to test the significance of differences. Different letters in the same row indicate significant differences (*p* < 0.05).

HNTs accelerated the accumulation of dry matter during the vegetative growth period and booting stage of wheat, and the HNT treatment had a higher dry weight than that with ATs (Figure 4). Biomass allocation differed in the wheat plants’ stem, leaf, and spike under the two different temperatures (Figure 5). At the heading stage, there was no significant difference in the stem or the leaf biomass of the same cultivar under the two temperatures. The stem biomass of JM22 increased significantly at anthesis under HNTs, but there was no significant difference between the spike and leaf biomass. For the stem, leaf, and spike biomass of JM44, there were no significant differences at anthesis under the two temperatures. At post-anthesis, only the spike biomass of JM22 decreased significantly under HNTs. At the grain-filling stage, there was no significant difference in the stem, leaf, and spike biomass of the same cultivar at the two temperatures. However, significant differences were observed in the stem, leaf, and spike biomass between the two cultivars at HNTs. The above results indicated that high temperatures accelerate JM22 and JM44 growth and development.

### 2.2. Interactive Impacts of HNTs and Genotypes on Physiological Processes in Wheat

**Figure 6:**
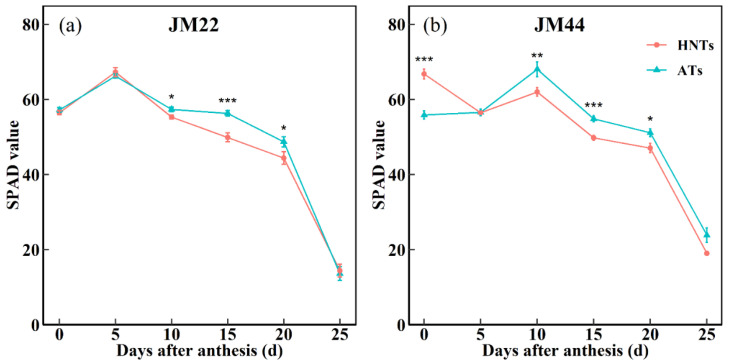
Chlorophyll content (SPAD value) of Jimai22 (JM22) (**a**)and Jimai44 (JM44) (**b**) at anthesis under the high nighttime temperatures (HNTs) and ambient temperatures (ATs). Analysis of variance (ANOVA) and the least significant difference (LSD) were used to test the significance of differences. *, **, and *** indicate a significant difference between HNTs and ATs at the 0.05, 0.01, and 0.001 levels, respectively.

The chlorophyll of the flag leaf is the main element involved in the photosynthesis of wheat, which directly affects the photosynthetic efficiency and the formation of yield [39]. The SPAD value of both wheat cultivars under the AT regime started to increase at post-anthesis and then decreased (Figure 6). Under the AT regime, the SPAD values peaked at 5 days after anthesis (DAA) in the JM22 leaves and was then reduced. However, JM44 leaves showed the highest SPAD values at 10 DAA and then decreased. Under the HNT regime, the maximum SPAD values were observed on JM22 leaves at 5 DAA and on JM44 leaves at anthesis. The SPAD values observed on JM22 and JM44 leaves under the HNT regime were lower than under the AT regime at 10 DAA, 15 DAA, and 20 DAA; the maximum variation in SPAD readings reached 11.4% at 15 DAA and 20.3% at anthesis, respectively. The maximum SPAD values observed on the two cultivars under different temperature regimes differed. The curves on JM22 and JM44 show that the SPAD value for both wheat cultivars decline faster under the HNT regime than the AT regime from 10 DAA to 20 DAA and from 0 DAA to 15 DAA, respectively. These results indicate that the flag leaf senescence occurred more quickly under the HNT regime.

There was a significant difference in the net photosynthetic rate of flag leaves between the two cultivars at 14 DAA under the AT regime (Table 1), with 14.37 ± 0.86 μmol m^−2^ s^−1^ for JM22 and 19.37 ± 0.18 μmol m^−2^ s^−1^ for JM44. The two wheat cultivars, however, did not show a significant difference in the net photosynthetic rate of flag leaves under two temperatures at 21 DAA. The net photosynthetic rate of flag leaves of JM22 at 7 DAA differed under different temperature regimes; it was 22.29 ± 0.85 μmol m^−2^ s^−1^ at HNTs and 19.52 ± 0.30 μmol m^−2^ s^−1^ at ATs. The two temperatures did not significantly affect the net photosynthetic rate of flag leaves at either 14 DAA or 21 DAA of JM22. For JM44, the net photosynthetic rate at 14 DAA differed between the HNT and AT regime, with 15.84 ± 0.72 μmol m^−2^ s^−1^ under the HNT regime and 19.37 ± 0.18 μmol m^−2^ s^−1^ under AT regime, with no difference between the two treatments at either 7 DAA or 21 DAA.

There was also no significant difference in the nighttime respiration rate of flag leaves of the two cultivars at ATs (Table 1). The nighttime respiration rates of flag leaves of the two cultivars at HNTs did not show significant differences at 7 DAA and 21 DAA. The nighttime respiration rates of flag leaves of JM22 at 14 DAA were 1.75 ± 0.16 μmol m^−2^ s^−1^ at HNTs and 0.90 ± 0.14 μmol m^−2^ s^−1^ at ATs. JM44 showed a significant increase in the nighttime respiration rate at 14 DAA, which is 1.33 ± 0.13 μmol m^−2^ s^−1^ at HNTs and 0.89 ± 0.11 μmol m^−2^ s^−1^ at ATs. The result showed that high nighttime temperatures mainly affect the net photosynthetic and nighttime respiration rates of flag leaves in the early stage after anthesis.

### 2.3. Interactive Impacts of HNTs and Genotype on Wheat Yields

**Figure 7:**
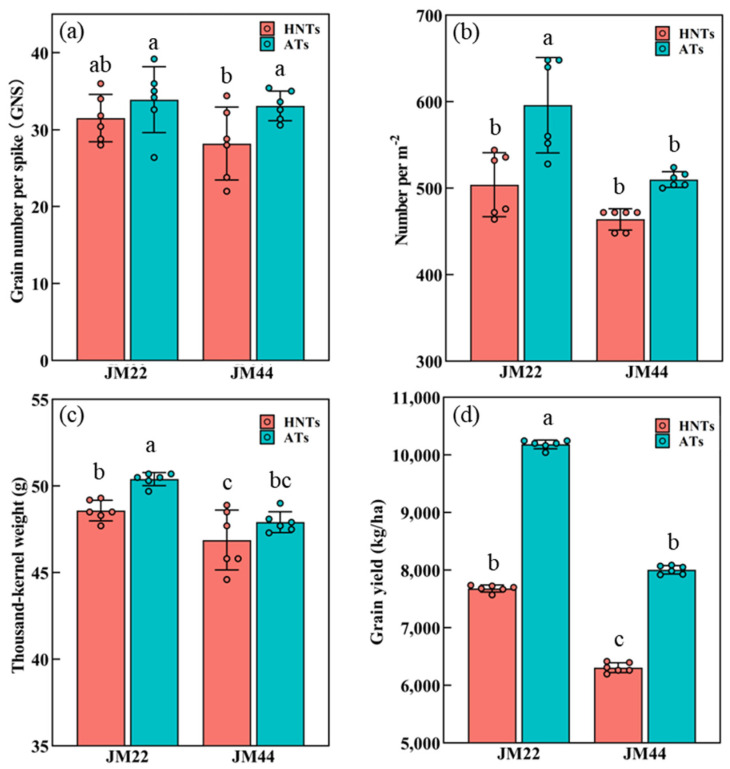
Number of spikes (NSs) (**a**), grain number per spike (GNS) (**b**), thousand-kernel weight (TKW) (**c**), grain yield (GY) (**d**) at harvest of Jimai22 (JM22) and Jimai44 (JM44) under high nighttime temperatures (HNTs) and ambient temperatures (ATs). Analysis of variance (ANOVA) and the least significant difference (LSD) were used to test the significance of differences. Different lower-case letters in the same column show significant differences (*p* < 0.05) within cultivars (or treatments).

Under the AT regime, JM22 showed a higher value for the number of fertile spikelets (NFSs), TKW, GY, and harvest index (HI), but a lower value for the number of sterile spikelets (NSSs) than JM44. There was no significant difference in NS and GNS between the two cultivars at ATs (Figure 7, Table S1). Increased temperatures reduced the NSs (from 596.0 ± 50.9 spikes per m^2^ to 504.0 ± 33.9 spikes per m^2^), TKW (from 50.4 ± 0.3 g to 48.6 ± 0.6 g), GY (from 10183.0 ± 69.3 kg/ha to 7681.0 ± 54.0 kg/ha), and the HI (from 41.5% to 34.2%) of JM22. There was no significant difference in the NFSs and GNS of JM22 between HNTs and ATs. Compared to ATs, NFSs decreased from 15.2 ± 0.6 to 13.3 ± 0.6, GNS decreased from 33.1 ± 1.2 to 28.2 ± 1.5, and NSSs increased from 3.6 ± 0.2 to 4.7 ± 0.4 of JM44 at HNTs. There was no significant difference in the NSs, TKW, and HI of JM44 under different regimes. Thus, increased temperatures reduced the GY of JM22 by 24.6% and JM44 by 21.2%. Their GYs under the two temperature regimes were in descending order as follows: JM22ATs > JM44ATs > JM22HNTs > JM44HNTs.

### 2.4. Interactive Impacts of HNTs and Genotype on Wheat Quality

The two wheat cultivars, JM44 and JM22, showed different quality parameters, which can be attributed to their different gluten strengths. Grown under the AT regime, JM22 exhibited a significantly lower protein content and gluten index, but higher starch and dry gluten content than JM44 (Table 2). The measurements of the quality traits of the two wheat cultivars showed they responded to HNTs in the same way except the dry gluten content. Increased temperatures reduced the flour’s starch content and gluten index. These results showed that high temperatures can reduce flour quality.

JM22 showed significantly lower trough viscosity, peak viscosity, and final viscosity than JM44 under the AT regime (Table 3). HNTs significantly reduced the peak viscosity and breakdown viscosity of the JM22 flour, but did not significantly affect the trough viscosity, final viscosity, setback viscosity, and pasting temperature of the JM22 flour. HNTs significantly reduced the breakdown viscosity, final viscosity, and setback viscosity of the JM44 flour, but did not significantly affect the trough viscosity, peak viscosity, and pasting temperature of the JM44 flour. The final viscosity and pasting temperature of the JM44 flour was not significantly different from that of JM22 under the HNT regime. Grown under the HNT regime, JM44 showed a significantly lower setback viscosity than JM22, while under the AT regime, there was no difference in setback viscosity between the two cultivars. The results suggested that the flour quality of JM22 was more stable than that of JM44 under high temperature conditions.

**Figure 8:**
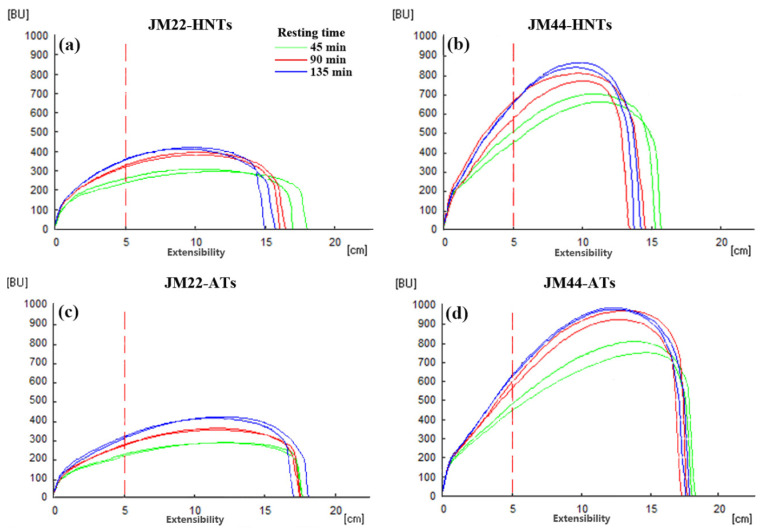
Tensile capacity of Jimai22 (JM22) (**a**,**c**) and Jimai44 (JM44) (**b**,**d**) dough under high nighttime temperatures (HNTs) and ambient temperatures (ATs). BU stands for tensile resistance.

The JM22 dough showed lower maximum resistance and tensile curve area at 45 min, 90 min, and 135 min of resting time than the JM44 dough under the AT regime (Figure 8). However, there was no difference in the extensibility between their doughs at two temperatures. The dough of the JM22 cultivar grown under the HNT regime showed a significantly lower extensibility and maximum resistance at the three resting time points in comparison with the AT regime. The dough of the JM44 cultivar grown under the HNT regime showed significantly lower extensibility, tensile curve area, and maximum resistance at three resting time points compared to the AT regime. The dough of the JM22 cultivar grown under the HNTs regime showed higher extensibility, lower tensile curve area, and maximum resistance than those of the JM44 cultivar at the three resting time points. Therefore, we inferred that dough of JM44 undergoes less variation than that of JM22 under high temperature conditions. The dough of JM22 grown under the AT regime showed significantly lower WA, DDT, ST, and FQN but significantly higher softening than JM44 (Table 4). For JM22, increased temperatures significantly increased water absorption, yet did not substantially affect DDT, ST, SD, and FQN. For JM44, there was no significant difference in the dough rheological properties between the AT and HNT regime, indicating that the quality properties of JM44 were more stable than those of JM22 under the two temperature regimes.

### 2.5. Principal Component Analysis (PCA) on the Yield and Quality Parameters of Wheat

**Figure 9:**
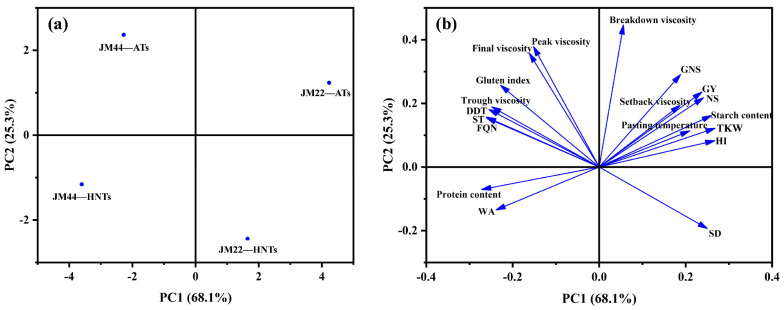
Principal component analysis (PCA) based on the yield and quality parameters of Jimai22 (JM22) and Jimai44 (JM44) under the high nighttime temperatures (HNTs) and ambient temperatures (ATs). (**a**) Loading plot; (**b**) score plot.

To identify the differences and similarities between the cultivar and treatment, and the interrelationships between the measured characteristics, the yield and quality parameters were subjected to PCA (Figure 9). The first and second principal components (PC1 and PC2) explained 68.1% and 25.3% of the overall variation, respectively. The distance between the locations of any two samples on the score plot is directly proportional to the degree of difference/similarity between them [40]. The distance between any two samples is large, indicating that the cultivar and treatment significantly affects the yield and quality of wheat. In addition, the correlations among the relative properties could be observed from the loading plot. The curves that are close to each other on the plot are positively correlated, while those in opposite directions are negatively correlated [40]. Among the yield-related parameters, GY and HI are positively correlated with NS, GNS, and TKW. For quality-related parameters, the trough viscosity is positively correlated with peak and final viscosity, and the DDT, ST, and FQN are positively correlated with gluten index. Moreover, the WA is positively correlated with protein content.

## 3. Discussion

### 3.1. Effects of HNTs on Morphology and Physiological Processes in Wheat

Temperature is an essential factor for crop growth and development, and temperature changes, particularly asymmetric changes, have been reported to impact crop growth and development significantly. In this study, HNTs significantly promoted the growth of the two wheat cultivars, and especially shortened the duration from the seedling stage to anthesis (Figure 1), which is consistent with the previous studies reporting that climate warming will significantly shorten the vegetative growth period of different wheat genotypes [9,12,41].

In general, the HNT treatment increased the tiller number and LAI at the early stage but decreased the tiller number and LAI at the late stage (Figure 2). This may be attributed to the fact that at the early stage, compared with ATs, HNTs raised the temperature and promoted the development of tillers and leaves [10]. However, because of the rapid development of tillers and leaves at the early stage, the wheat consumed a lot of nutrients, resulting in nutrient deficiency and reduced effective tillers and leaves at the late stage. In addition, the HNT treatment sped up the developmental process of wheat; there was not enough time for the late developed tillers to complete spike development, resulting in the reduction in the tiller number and LAI at the late stage. Different from the previous report showing that warming increased biomass at the maturity stage [12,42], the current results demonstrated that the HNT regime does not affect biomass at the grain-filling and maturity stages (Figure 4). This may be related to the experiment’s geographical location, the method of warming, and the wheat cultivar. The early rapid development and growth may cause a reduction in the NFSs of wheat, given that jointing and pre-anthesis are the critical developmental stages determining the sink strength (i.e., the number of spikelets per spike) [23]; the shortened stages led to the incomplete development of florets, and the early occurrence of the advanced stages increased the chance of florets undergoing stress at low temperatures in spring [43].

Plant height is a critical determinant of yield [44,45]. HNTs significantly increased the plant height in the current study (Figure 3a,b). A further analysis of the main stems found that the second basal internode and peduncle length were significantly increased in both cultivars (Figure 3c,d). Given that the increase in plant height would reduce the distribution of assimilate to the spike, reduce the fertility of the spike, and thus reduce the yield [23], the second basal internode has a significant negative correlation with yield [46]. Therefore, the increase in the plant height and second basal internode under HNTs may lead to a lower yield (Figure 7). The biomass at anthesis was significantly correlated with the GY. The biomass of the wheat stem and leaf post-anthesis in the two cultivars under the HNT regime decreased more quickly compared with ATs, while the biomass of spikes increased more slowly under the HNT regime. This may be attributed to the accelerated senescence and reduced NFSs per spike. Therefore, the current results indicated that HNTs negatively affected the dry matter partitioning in the two wheat cultivars.

The net photosynthetic rates of flag leaves of JM44 were significantly lower under the HNT regime than those under the AT regime at 14 DDA (Table 1). This may be due to the fact that wheat responds differently to HNTs at various stages, and the effect at the post-anthesis may be greater than that at the overwintering stage [47]. The nighttime respiration rates of the two wheat cultivars increased under the HNT regime compared with ATs, which increased carbon consumption in wheat (Table 1), agreeing with the previously reported results [48]. Excessive carbon consumption also contributed to the slow accumulation of dry matter post-anthesis [49]. The SPAD values of the flag leaf of both wheat cultivars under the HNT regime decreased more quickly than those under AT regime post-anthesis (Figure 6). These results indicated that HNTs caused accelerated leaf senescence, reduced the photosynthetic rate, and increased the respiration rate in the two wheat cultivars during the grain-filling stage.

### 3.2. Effects of HNTs on Wheat Yield

In the present experiment, HNTs significantly decreased the NSs and TKW of JM22, and decreased the NFSs and GNS of JM44 (Figure 7, Table S1). Similar results have been reported that the number of grains were decreased by 6% for each °C increase in nighttime temperature [11]; the current results also demonstrated that the NSSs increased significantly in the two wheat cultivars under the HNT regime (Table S1).

The HNT regime allows the allocation and partitioning of more assimilates to the growing juvenile spikes before anthesis, and florets continue to develop with many floret primordia initiating, leading to a shortage of assimilates for weak later-initiating floret primordia to abort [23]. The higher floret primordial mortality resulted in a low tillering spike rate and NFSs (Table S1), agreeing with the previously reported result [50]. In addition, the HNT treatment significantly reduced SPAD values during the grain-filling stage, resulting in the early termination of assimilates transported to grains, and thus a lower starch content in grains and less TKW. JM22 and JM44 under the HNT regime yielded 24.6% and 21.2% less grains than under the AT regime (Figure 7). The decreased GY can be attributed to the decreased sink strength in the vegetative growth phase, and reduced photosynthate accumulation in the reproductive growth period [23]. Compared with the AT regime, the HNT regime reduced the HI of JM22, but there was no difference in the HI of JM44 under both regimes (Table S1).

Worldwide, a general consensus is that increased temperatures can reduce the wheat growing season and GY if no adaptation strategies are adopted [51]. However, a previous study showed that increasing the temperature up to 3 °C increases winter wheat GY while decreases spring wheat GY in China through field warming experiments at multiple sites [15]. There were spatio-temporal differences in the response of winter wheat GY and phenology to climate warming and differences in temperature and precipitation patterns in different production areas, which may explain the inconsistencies among experiments. However, with the current increase in global temperature, the negative effect of HNTs on wheat GY and quality may intensify [9,52].

### 3.3. Effects of HNTs on Wheat Quality

The grain protein and starch contents are crucial characteristics affecting not only nutritional quality but also the processing quality of wheat flour [53]. The effects of HNTs on wheat grain quality were almost consistent, that is, HNTs increased the protein content and decreased the starch content of wheat grains (Table 2). This may be attributed to an earlier cessation of starch accumulation in wheat grains under the HNT regime [54], and a corresponding increase in protein content [50,55]. Not surprisingly, TKW was positively correlated with starch content but negatively correlated with protein content (Figure 9), which further explains that the decrease in starch accumulation under the HNT treatment resulted in the decrease of TKW and GY. Even though the protein content was increased under HNTs, the gluten index showed a significant reduction for the two wheat cultivars (Table 2), which may be related to the reduced ratio of glutenin to gliadin [56].

Compared with the AT regime, HNTs reduced the peak viscosity and final viscosity of wheat flour due to the reduced starch content of the flour (Table 3), as peak viscosity and final viscosity were positively correlated with starch content [57]. Compared with the AT regime, HNTs reduced the dough tensile properties of both cultivars (Figure 8). In general, the protein content played a positive role. However, some opposite results suggest that protein composition and starch content may play a more critical role than protein content, which reduces tensile properties. HNTs increased the water absorption of JM22 flour but there was no difference in JM44 flour (Table 4). The change in water absorption can be attributed to the different contributions of gluten protein, gluten–starch interactions, and dough network structure [57]. Starch can be a dominant factor affecting the dough properties of wheat cultivars with a genetic background of weak gluten strength [58,59]. Gluten strength has been considered the dominant factor affecting dough properties for wheat varieties with strong gluten backgrounds, and the DDT, ST, and FQN are positively correlated with the gluten index (Figure 9). JM44 exhibited higher DDT, ST, and FQN than JM22 did, which can be attributed to the higher gluten index of JM44. The HNT treatment significantly reduced the gluten index of the two cultivars, but had no significant effect on DDT, ST, and FQN, which could be attributed to the effects of the gluten–starch interaction and the dough network structure on dough characteristics. Future studies may focus on explaining this change in terms of the gluten–starch interaction and dough network structure.

### 3.4. Climate Change Impacts on Wheat Production in the Future

Adapting crop yield and quality to climate change is an ongoing challenge for food security and environmental sustainability [60]. In such cases, characterizing crop responses to environmental changes provides valuable information for designing adaptation strategies. A study in India showed that wheat could adapt to increased HNTs during the vegetative stage, but at the filling stage, HNTs had more effects on yield components. Even shorter HNTs significantly reduced yield [61]. An experiment in China showed that the HNT regime performed in winter promoted pre-anthesis wheat growth and leaf development, improved post-anthesis photosynthetic capacity, and ultimately increased GY [10]. An experiment in Argentina showed that the HNT regime performed from the jointing stage to 10 DAA reduced GY by about 7% for each 1 °C increase in nighttime temperature in well-adapted wheat and barley cultivars [17]. A study in the United States showed that the HNT regimes performed between 10 DAA and physiological maturity significantly reduced the yield in 11 of 12 wheat cultivars [19]. All the above experiments were conducted in the field, and there were differences in HNT regimes performed at different stages. However, given that HNTs occur during the whole growth period of wheat, the impact of HNTs on future crops, either positive or negative, may be exaggerated. Therefore, it is necessary to conduct field HNT experiments with more wheat varieties over the whole growth period. Information on HNTs influencing quality is limited, and a field experiment showed that the HNT regime at post-anthesis negatively affected the wheat’s starch, protein, and micronutrient content [62]. HNTs have been reported to reduce starch content but increase protein and lipid contents in a controlled environment [55]. Climate warming may have different effects on different growth periods of crops, all of which ultimately have a combined effect on their yield and quality. These effects need to be fully evaluated in further research and changes in agricultural planting strategies would be well considered by policy makers.

## 4. Materials and Methods

### 4.1. Experimental Site

Field experiments were conducted at Licheng Experiment Station of Shandong Academy of Agricultural Sciences (36°42′ N, 117°12′ E), Shandong Province, China, during a growing season (2021–2022) of winter wheat. The soil type in the test plot is brown loam with total N, P, and K contents of 135.0 mg/kg, 25.6 mg/kg and 209.0 mg/kg, respectively and a soil capacity of 1.13 g/cm^3^.

### 4.2. Experimental Design

Seeds were sown in October 2021, and wheat grains were harvested in June 2022. The experiment was set up in a randomized block design under the HNT treatment and control at ATs, with cultivars and treatments replicated twice. Each repeat contains three biological replicates. A passive infrared reflective heating device was used for the HNT treatment. This device works on the principle that the heated ground releases heat energy radiated outward in the form of infrared rays due to the temperature difference between the ground and the air at night. The role of the polymer film is to reflect the infrared energy, reducing the loss of heat and keeping the ground warmed. The height of the polymer film is adjustable utilizing a wheel kept at a distance of 20 cm above wheat. Canopy temperature was recorded using a remote temperature and humidity collection instrument (S20A-2300, Xuzhou Fala Electronic Technology Co., Ltd., Xuzhou, Jiangsu, China) every 30 min (Figure S1). For the sake of simplicity and clarity in this study, we use HNTs to represent high temperatures and ATs to represent the ambient temperatures.

Cultivars JM22 and JM44 were used as experimental materials. JM22 is a high-yielding and medium-gluten wheat cultivar, and JM44 is a strong-gluten wheat cultivar. They show significant differences in genotype, growth period, yield, and flour quality. Each planting plot was 6 m^2^, and the planting density was 300 plants per m^2^, with 25 cm row spacing. Doses of 240 kg N hm^−2^, 210 kg P_2_O_5_ hm^−2^, and 300 kg K_2_O hm^−2^ were applied as basal fertilizer before sowing, and another 240 kg N hm^−2^ was applied for top-dressing at the jointing and booting stages. Nitrogenous fertilizer was used as urea (46% N). Irrigation was applied at an amount of 600 m^3^ per hectare during the jointing stage.

### 4.3. Measurements of Wheat Leaf Age and Tiller Number

Ten to fifteen individual wheat plants were sampled for each treatment. The wheat leaf age was measured at the over-wintering stage, and the tiller number was counted at the over-wintering, re-greening, jointing, booting, heading, anthesis, grain-filling, and maturity stages.

### 4.4. Determination of Leaf Area and LAI

Ten to fifteen individual wheat plants were selected for each treatment. The maximum length and width of each leaf was measured to calculate the wheat leaf area (cm^2^), total leaf area per plant (cm^2^), and LAI (m^2^/m^2^) according to the following formula.

Leaf area = 0.77 × leaf length × leaf width

Total leaf area per plant = Σ (Leaf area)

LAI = average total leaf area per plant × planting density

### 4.5. Measurements of Wheat Plant Height and Internode Length of Main Stem

Ten to fifteen individual wheat plants were selected for each treatment. The wheat plant height was measured at over-wintering, re-greening, jointing, booting, heading, anthesis, grain-filling, and maturity stages. The internode length of the main stem was measured at the maturity stage.

### 4.6. Determination of Above-Ground Biomass

Ten to fifteen individual wheat plants were selected for each treatment. To calculate biomass distribution at heading, anthesis, post-anthesis, and grain-filling stages, the above-ground biomass of wheat was divided into three organs: stem, leaf, and spike, and their biomass was dried at 80 °C for at least 48 h and weighed. For other growth periods, the above-ground organ was sampled directly, and the biomass was dried at 80 °C for at least 48 h and weighed.

### 4.7. Chlorophyll Content Measurement

Relative chlorophyll content was measured using SPAD-502 (Minolta Camera Co., Osaka, Japan) on each flag leaf of 10 individual wheat plants and mean values were calculated.

### 4.8. Determination of Photosynthetic Parameters and Nighttime Respiration Rate

Leaf gas exchange was measured using a portable photosynthesizer (LI-6400XT; Li-Cor, Lincoln, NE, USA). Gas exchange measurements were recorded every seven days at post-anthesis. Three wheat flag leaves per plot were selected for gas exchange measurements in the morning (9:00–11:00 a.m.) and evening (8:00–10:00 p.m.). The net photosynthetic rate of flag leaves was determined, and the net photosynthetic light flux density was 1000 μmol m^−2^ s^−1^. Since photosynthesis was not performed at night, the nighttime net photosynthetic rate was regarded as respiration.

### 4.9. Measurements of Yield in Wheat

After physiological maturity, 1 m^2^ of the grown wheat area was selected to determine the NS per m^2^, the GNS, the NFSs per spike, and the NSSs per spike. The wheat was harvested from each m^2^ plot, and the GY and harvest index (HI, calculated as GY divided by above-ground biomass) were determined. TKW was measured after the grains were dried in the oven for 48 h to maintain a consistent weight.

### 4.10. Determination of Wheat Flour Quality

The grains of the two wheat cultivars harvested and stored for two months were ground in a small pilot mill (Brabender, Duisburg, Germany) with a total flour yield of 60%. The flour protein and starch content were determined using an Infratec1241 NIR profiler (Foss, Copenhagen, Denmark). According to the reported method [63], dry gluten content and gluten index were measured using the GM2200 gluten meter (Perten, Stockholm, Sweden). Peak viscosity, trough viscosity, final viscosity, setback viscosity, and breakdown viscosity were measured using the RVA-4 rapid visco analyzer (Newport, Melbourne, Australia) following the method of AACC 22-08.02 [3]. The tensile resistance, extension degree, and stretching ratio were measured by the process of AACC 54-10.01 using the Extensograph-E automatic powder analyzer (Brabender, Duisburg, Germany) [3]. WA, DDT, ST, softening degree (SD), and farinograph quality number (FQN) were measured according to AACC 54-21.02 using the Farinograph-AT automatic powder analyzer (Brabender, Duisburg, Germany) [3].

### 4.11. Statistical Analysis

The experimental data were analyzed using Microsoft Office Excel 2016, and statistical results and graphs were produced using R4.1.2 [64]. Analysis of variance (ANOVA) and least significant difference (LSD) tests were used to test the significance of differences between treatments and between different developmental stages. Principal component analysis (PCA) based on the yield and quality parameters was performed using the OriginPro software (v. 2021, Originlab, Northampton, MA, USA). The results are presented as mean ± Standard error of mean.

## 5. Conclusions

The effect of HNTs on the growth, physiological processes, yield, and quality of the two wheat cultivars was deeply investigated in this study. HNTs shortened the growth stage of wheat, especially the vegetative growth stage, and led to early anthesis in the grain. The HNT treatment hindered the development of flower organs and reduced the GNS. At the grain-filling stage, HNTs accelerated leaf senescence and reduced TKW. The decrease in GY under the HNT treatment is attributed to the adverse effect of high temperature on growth and development. Interestingly, the GY of JM44 decreased less, indicating its stronger ability to resist heat stress. Regarding quality, HNTs increased the protein content, and decreased the starch content gluten index, starch pasting characteristics, dough stability time, and tensile properties.

In summary, HNTs significantly shortened seedling emergence and anthesis periods in this study. In addition, HNTs increased the respiration rate at anthesis and impeded wheat pollination and grain maturity. HNTs also accelerated leaf senescence, and decreased GY. Taken together, HNTs negatively influenced the flour quality of the two wheat cultivars. Our results elucidate the mechanism of action of the HNT treatment on wheat yield and quality and provide a solid argument for the need to understand the responses and adaptation of crop production to climate change. It is necessary engage in in-depth studies using transcriptomic and metabolomic analyses in the future so that the effect of high nighttime temperatures on the growth, yield, and quality of wheat can be further clarified.

## Figures and Tables

**Table 1:** Effect of high nighttime temperatures on net photosynthetic and nighttime respiration rates of flag leaves of Jimai22 (JM22) and Jimai44 (JM44).

Cultivar	Treatment	Net Photosynthetic Rate (μmol m^−2^ s^−1^)			Nighttime Respiration Rate (μmol m^−2^ s^−1^)		
		7 DAA	14 DAA	21 DAA	7 DAA	14 DAA	21 DAA
JM22	HNTs	22.29 ± 0.85 a	13.69 ± 1.38 c	9.69 ± 0.87 a	1.54 ± 0.15 a	1.75 ± 0.16 a	0.83 ± 0.09 a
	ATs	19.52 ± 0.30 b	14.37 ± 0.86 c	11.41 ± 0.57 a	1.19 ± 0.12 ab	0.90 ± 0.14 c	0.67 ± 0.03 a
JM44	HNTs	20.16 ± 0.63 b	15.84 ± 0.72 b	9.92 ± 1.01 a	1.07 ± 0.06 ab	1.33 ± 0.13 b	0.39 ± 0.07 a
	ATs	19.43 ± 0.44 b	19.37 ± 0.18 a	10.99 ± 0.25 a	0.82 ± 0.26 b	0.89 ± 0.11 c	0.68 ± 0.25 a

HNTs, high nighttime temperatures; ATs, ambient temperatures; DAA, days after anthesis. Analysis of variance (ANOVA) and the least significant difference (LSD) were used to test the significance of differences. Different lower-case letters in the same column show significant differences (*p* < 0.05) within cultivars (or treatments).

**Table 2:** Effect of high nighttime temperatures on flour quality of Jimai22 (JM22) and Jimai44 (JM44).

Cultivar	Treatment	Moisture (%)	Protein Content (%)	Starch Content (%)	Gluten Index (%)	Dry Gluten Content (%)	Whiteness (%)
JM22	HNTs	10.5 ± 0.3 a	14.7 ± 0.3 bc	57.1 ± 0.2 b	50.7 ± 10.3 d	13.8 ± 0.9 a	77.6 ± 0.9 a
	ATs	10.7 ± 0.6 a	14.1 ± 0.3 c	57.6 ± 0.1 a	61.8 ± 8.7 c	12.4 ± 1.0 b	78.7 ± 0.9 a
JM44	HNTs	10.5 ± 0.4 a	16.0 ± 0.5 a	56.6 ± 0.2 c	87.8 ± 0.1 b	11.3 ± 0.7 bc	76.2 ± 2.9 b
	ATs	10.4 ± 0.2 a	15.3 ± 0.8 ab	57.0 ± 0.2 b	97.2 ± 1.3 a	10.9 ± 0.7 c	76.7 ± 3.6 ab

HNTs, high nighttime temperatures; ATs, ambient temperatures. Analysis of variance (ANOVA) and the least significant difference (LSD) were used to test the significance of differences. Different lower-case letters in the same column show significant differences (*p* < 0.05) within cultivars (or treatments).

**Table 3:** Effect of high nighttime temperatures on flour pasting properties of Jimai22 (JM22) and Jimai44 (JM44).

Cultivar	Treatment	Trough Viscosity (cP)	Peak Viscosity (cP)	Breakdown Viscosity (cP)	Final Viscosity (cP)	Setback Viscosity (cP)	Pasting Temperature (°C)
JM22	HNTs	1577.3 ± 30.2 b	1804.0 ± 81.5 c	226.7 ± 57.2 c	2894.0 ± 91.0 b	1316.7 ± 86.6 a	71.8 ± 7.0 a
	ATs	1647.5 ± 30.3 b	2141.0 ± 83.1 b	493.5 ± 75.3 ab	2989.9 ± 41.1 b	1342.4 ± 18.7 a	80.1 ± 3.2 a
JM44	HNTs	1967.7 ± 88.3 a	2229.3 ± 141.2 ab	261.7 ± 53.8 bc	3052.0 ± 64.6 b	1084.3 ± 27.1 b	72.6 ± 6.6 a
	ATs	1972.8 ± 30.1 a	2493.9 ± 59.7 a	521.1 ± 56.0 a	3309.1 ± 40.1 a	1336.4 ± 26.8 a	70.8 ± 3.1 a

HNTs, high nighttime temperatures; ATs, ambient temperatures; cP indicates centipoise. Analysis of variance (ANOVA) and the least significant difference (LSD) were used to test the significance of differences. Different lower-case letters in the same column show significant differences (*p* < 0.05) within cultivars (or treatments).

**Table 4:** Effect of high nighttime temperatures on dough rheological properties of Jimai22 (JM22) and Jimai44 (JM44).

Cultivar	Treatment	MT (min)	DDT (min)	WA (%)	ST (min)	SD (ICC) (FE)	FQN (mm)
JM22	HNTs	20	3.05 ± 0.29 b	65.20 ± 4.84 a	4.05 ± 1.15 b	100.00 ± 3.51 a	59.33 ± 8.00 b
	ATs	20	3.19 ± 1.12 b	59.68 ± 4.43 b	4.25 ± 1.72 b	91.13 ± 7.26 a	57.38 ± 7.50 b
JM44	HNTs	45	22.45 ± 3.12 a	66.10 ± 0.70 a	17.95 ± 0.88 a	35.56 ± 8.31 b	347.00 ± 21.63 a
	ATs	45	26.07 ± 1.85 a	66.00 ± 0.91 a	18.39 ± 1.19 a	30.00 ± 5.29 b	366.56 ± 23.21 a

HNTs, high nighttime temperatures; ATs, ambient temperatures; MT, measuring time; DDT, dough development time; WA, water absorption; ST, stability time; SD (ICC), softening degree (ICC/12 min after max.); FQN, farinograph quality number. Analysis of variance (ANOVA) and the least significant difference (LSD) were used to test the significance of differences. Different lower-case letters in the same column show significant differences (*p* < 0.05) within cultivars (or treatments).

## Data Availability

The original contributions presented in the study are included in the article/Supplementary Materials; further inquiries can be directed to the corresponding authors.

## Supplementary Materials

The following supporting information can be downloaded at: https://www.mdpi.com/article/10.3390/plants13213071/s1, Figure S1. Differences in monthly mean nighttime temperatures between high nighttime temperatures (HNTs) and ambient temperatures (ATs). Figure S2. Leaf age of Jimai22 (JM22) and Jimai44 (JM44) at overwintering stage under the high nighttime temperatures (HNTs) and ambient temperatures (ATs). Analysis of variance (ANOVA) and the least significant difference (LSD) were used to test the significance of differences. **, and *** indicate a significant difference between HNTs and ATs at the 0.01, and 0.001 levels, respectively. Table S1. Effect of high nighttime temperatures on yield indices at harvest of Jimai22 (JM22) and Jimai44 (JM44).

## Abbreviations

The following abbreviations are used in this manuscript:

JM22	Jimai22
JM44	Jimai44
HNTs	High nighttime temperatures
ATs	Ambient temperatures
GY	Grain yield
LAI	Leaf area index
HI	Harvest index
TKW	Thousand-kernel weight
DAA	Days after anthesis
NS	Number of spikes
GNS	Grain number per spike
WA	Water absorption
DDT	Dough development time
ST	Stability time
NFS	Number of fertile spikelets
NSS	Number of sterile spikelets
SD	Softening degree
FQN	Farinograph quality number
LSD	Least significant difference
PCA	Principal component analysis
ANOVA	Analysis of variance
CP	Centipoise

## References

[1] FAO (Food and Agriculture Organization). https://www.fao.org/faostat/en/.

[2] WMO (World Meteorological Organization) The State of the Global Climate 2021. https://public.wmo.int/en/our-mandate/climate/wmo-statement-state-of-global-climate.

[3] AACC (American Association of Cereal Chemists) (2011). Approved Methods of the American Association of Cereal Chemists.

[4] Prasad P.V.V., Bheemanahalli R., Jagadish S.V.K. (2017). Field crops and the fear of heat stress-opportunities, challenges and future directions. Field Crops Res..

[5] Shew A.M., Tack J., Nalley L.L., Chaminuka P. (2020). Yield reduction under climate warming varies among wheat cultivars in South Africa. Nat. Commun..

[6] Alexander L.V., Zhang X., Peterson T.C., Caesar J., Gleason B., Klein T.A.M.G., Haylock M., Collins D., Trewin B., Rahimzadeh F. (2006). Global observed changes in daily climate extremes of temperature and precipitation. J. Geophys. Res..

[7] Sillmann J., Kharin V.V., Zhang X., Zwiers F.W., Bronaugh D. (2013). Climate extremes indices in the CMIP5 multimodel ensemble: Part 1. Model evaluation in the present climate. J. Geophys. Res. Atmos..

[8] Jagadish S.V., Murty M.V., Quick W.P. (2015). Rice responses to rising temperatures-challenges, perspectives and future directions. Plant Cell Environ..

[9] Tao F., Zhang L., Zhang Z., Chen Y. (2022). Climate warming outweighed agricultural managements in affecting wheat phenology across China during 1981–2018. Agric. For. Meteorol..

[10] Fan Y., Tian M., Qin B., Jiang Q., Tian Z., Han H., Jiang Q., Cao W., Dai T. (2015). Winter night warming improves pre-anthesis crop growth and post-anthesis photosynthesis involved in grain yield of winter wheat (*Triticum aestivum* L.). Field Crops Res..

[11] García G.A., Dreccer M.F., Miralles D.J., Serrago R.A. (2015). High night temperatures during grain number determination reduce wheat and barley grain yield: A field study. Glob. Chang. Biol..

[12] Li Y., Hou R., Tao F. (2020). Interactive effects of different warming levels and tillage managements on winter wheat growth, physiological processes, grain yield and quality in the North China Plain. Agric. Ecosyst. Environ..

[13] Yadav M.R., Choudhary M., Singh J., Lal M.K., Jha P.K., Udawat P., Gupta N.K., Rajput V.D., Garg N.K., Maheshwari C. (2022). Impacts, tolerance, adaptation, and mitigation of heat stress on wheat under changing climates. Int. J. Mol. Sci..

[14] Zheng C., Zhang J., Chen J., Chen C., Tian Y., Deng A., Song Z., Nawaz M.M., van-Groenigen K.J., Zhang W. (2017). Nighttime warming increases winter-sown wheat yield across major Chinese cropping regions. Field Crops Res..

[15] He D., Fang S., Liang H., Wang E., Wu D. (2020). Contrasting yield responses of winter and spring wheat to temperature rise in China. Environ. Res. Lett..

[16] Narayanan S., Prasad P.V.V., Fritz A.K., Boyle D.L., Gill B.S. (2015). Impact of high nighttime and high daytime temperature stress on winter wheat. J. Agron. Crop Sci..

[17] García G.A., Serrago R.A., Dreccer M.F., Miralles D.J. (2016). Post-anthesis warm nights reduce grain weight in field-grown wheat and barley. Field Crops Res..

[18] Zhao C., Liu B., Piao S., Wang X., Lobell D.B., Huang Y., Huang M., Yao Y., Bassu S., Ciais P. (2017). Temperature increase reduces global yields of major crops in four independent estimates. Proc. Natl. Acad. Sci. USA.

[19] Hein N.T., Wagner D., Bheemanahalli R., Šebela D., Bustamante C., Chiluwal A., Neilsen M.L., Jagadish S.V.K. (2019). Integrating field-based heat tents and cyber-physical system technology to phenotype high night-time temperature impact on winter wheat. Plant Methods.

[20] Fan Y., Qin B., Yang J., Ma L., Cui G., He W., Yu T., Zhang W., Ma S., Ma C. (2024). Night warming increases wheat yield by improving pre-anthesis plant growth and post-anthesis grain starch biosynthesis. J. Integr. Agric..

[21] Kang X., Hou R., Yang G. (2024). Effects of climactic warming on the starch and protein content of winter wheat grain under conservation tillage in the North China Plain. Soil Till. Res..

[22] Sierra-Gonzalez A., Molero G., Rivera-Amado C., Babar M.A., Reynolds M.P., Foulkes M.J. (2021). Exploring genetic diversity for grain partitioning traits to enhance yield in a high biomass spring wheat panel. Field Crops Res..

[23] Slafer G.A., Foulkes M.J., Reynolds M.P., Murchie E.H., Carmo-Silva E., Flavell R., Gwyn J., Sawkins M., Griffiths S. (2023). A ‘wiring diagram’ for sink strength traits impacting wheat yield potential. J. Exp. Bot..

[24] Kuktaite R., Ravel C., Igrejas G., Ikeda T., Guzmán C. (2020). Wheat gluten protein structure and function: Is there anything new under the sun?. Wheat Quality for Improving Processing and Human Health.

[25] Aono S., Nishitsuji Y., Iwaki S., Hayakawa K. (2024). Effects of environmental temperature during maturation on protein characteristics in spring wheat (*Triticum aestivum* cv. Haruyokoi). J. Cereal Sci..

[26] Li S., Wang J., Ding M., Min D., Wang Z., Gao X. (2019). The influence of night warming treatment on the micro-structure of gluten in two wheat cultivars. Food Res. Int..

[27] Peterson C.J., Graybosch R.A., Shelton D.R., Baenziger P.S. (1998). Baking quality of hard winter wheat: Response of cultivars to environment in the Great Plains. Euphytica.

[28] Békés F. (2012). New aspects in quality related wheat research: 1. Challenges and achievements. Cereal Res. Commun..

[29] Hernández-Espinosa N., Mondal S., Autrique E., Gonzalez-Santoyo H., Crossa J., Huerta-Espino J., Singh R.P., Guzmán C. (2018). Milling, processing and end-use quality traits of CIMMYT spring bread wheat germplasm under drought and heat stress. Field Crops Res..

[30] Haddad L., Hawkes C., Webb P., Thomas S., Beddington J., Waage J., Flynn D. (2016). A new global research agenda for food. Nature.

[31] Lin Z., Chang X., Wang D., Zhao G., Zhao B. (2015). Long-term fertilization effects on processing quality of wheat grain in the North China Plain. Field Crops Res..

[32] Battenfield S.D., Guzmán C., Gaynor R.C., Singh R.P., Peña R.J., Dreisigacker S., Fritz A.K., Poland J.A. (2016). Genomic selection for processing and end-use quality traits in the CIMMYT spring bread wheat breeding program. Plant Genome.

[33] Mastilović J., Živančev D., Lončar E., Malbaša R., Hristov N., Kevrešan Ž. (2017). Effects of high temperatures and drought during anthesis and grain filling period on wheat processing quality and underlying gluten structural changes. J. Sci. Food Agric..

[34] Zi Y., Shen H., Dai S., Ma X., Ju W., Wang C., Guo J., Liu A., Cheng D., Li H. (2019). Comparison of starch physicochemical properties of wheat cultivars differing in bread-and noodle-making quality. Food Hydrocoll..

[35] Zhu J., Huang S., Khan K., O»Brien L. (2001). Relationship of protein quantity, quality and dough properties with Chinese steamed bread quality. J. Cereal Sci..

[36] Dencic S.S., Mladenov N., Kobiljski B.D.J. (2012). Effects of genotype and environment on breadmaking quality in wheat. Int. J. Plant Prod..

[37] Li Q., Liu R., Wu T., Zhan M. (2017). Interactions between soluble dietary fibers and wheat gluten in dough studied by confocal laser scanning microscopy. Food Res. Int..

[38] Russell K., Van-Sanford D.A. (2020). Breeding wheat for resilience to increasing nighttime temperatures. Agronomy.

[39] Yang B., Chen N., Dang Y., Wang Y., Wen H., Zheng J., Zheng X., Zhao J., Lu J., Qiao L. (2022). Identification and validation of quantitative trait loci for chlorophyll content of flag leaf in wheat under different phosphorus treatments. Front. Plant Sci..

[40] Gao L., Van B.F., Lewille B., Haesaert G., Eeckhout M. (2023). Characterization and comparative study on structural and physicochemical properties of buckwheat starch from varieties. Food Hydrocoll..

[41] Wang J., Wang E., Feng L., Yin H., Yu W. (2013). Phenological trends of winter wheat in response to varietal and temperature changes in the North China Plain. Field Crops Res..

[42] Fang S., Cammarano D., Zhou G., Tan K., Ren S. (2015). Effects of increased day and night temperature with supplemental infrared heating on winter wheat growth in North China. Eur. J. Agron..

[43] Ji H., Xiao L., Xia Y., Song H., Liu B., Tang L., Cao W., Zhu Y., Liu L. (2017). Effects of jointing and booting low temperature stresses on grain yield and yield components in wheat. Agric. For. Meteorol..

[44] Richards R.A. (1992). The effect of dwarfing genes in spring wheat in dry environments, 1. Agronomic characteristics. J. Agric. Res..

[45] Flintham J.E., Börner A., Worland A.J., Gale M.D. (1997). Optimizing wheat grain yield: Effects of *Rht* (gibberellin-insensitive) dwarfing genes. J. Agric. Sci..

[46] Rivera-Amado C., Trujillo-Negrellos E., Molero G., Reynolds M.P., Sylvester-Bradley R., Foulkes M.J. (2019). Optimizing dry-matter partitioning for increased spike growth, grain number and harvest index in spring wheat. Field Crops Res..

[47] Fan Y., Tian Z., Yan Y., Hu C., Abid M., Jiang D., Ma C., Huang Z., Dai T. (2017). Winter night-warming improves post-anthesis physiological activities and sink strength in relation to grain filling in winter wheat (*Triticum aestivum* L.). Front. Plant Sci..

[48] Peng S., Piao S., Ciais P., Myneni R.B., Chen A., Chevallier F., Dolman A.J., Janssens I.A., Peñuelas J., Zhang G. (2013). Asymmetric effects of daytime and night-time warming on Northern Hemisphere vegetation. Nature.

[49] Joshi J., Amthor J.S., McCarty D.R., Messina C.D., Wilson M.A., Harvey M.A., Hanson A.D. (2023). Why cutting respiratory CO_2_ loss from crops is possible, practicable, and prudential. Mod. Agric..

[50] Impa S.M., Sunoj V.S.J., Krassovskaya I., Bheemanahalli R., Obata T., Jagadish S.V.K. (2019). Carbon balance and source-sink metabolic changes in winter wheat exposed to high nighttime temperature. Plant Cell Environ..

[51] Minoli S., Müller C., Elliott J., Ruane A.C., Jägermeyr J., Zabel F., Dury M., Folberth C., François L., Hank T. (2019). Global response patterns of major rainfed crops to adaptation by maintaining current growing periods and irrigation. Earth’s Future.

[52] Ren S., Qin Q., Ren H. (2019). Contrasting wheat phenological responses to climate change in global scale. Sci. Total Environ..

[53] Asseng S., Martre P., Maiorano A., Rötter R.P., O’Leary G.J., Fitzgerald G.J., Girousse C., Motzo R., Giunta F., Babar M.A. (2019). Climate change impact and adaptation for wheat protein. Glob. Chang. Biol..

[54] Hurkman W.J., McCue K.F., Altenbach S.B., Korn A.M., Tanaka C.K., Kothari K.M., Johnson E.L., Bechtel D.B., Wilson J.D., Anderson O.D. (2003). Effect of temperature on expression of genes encoding enzymes for starch biosynthesis in developing wheat endosperm. Plant Sci..

[55] Impa S.M., Vennapusa A.R., Bheemanahalli R., Sabela D., Boyle D., Walia H., Jagadish S.V.K. (2020). High night temperature induced changes in grain starch metabolism alters starch, protein, and lipid accumulation in winter wheat. Plant Cell Environ..

[56] Corbellini M., Canevar M.G., Mazza L., Ciaffi M., Lafiandra D., Borghi B. (1997). Effect of the duration and intensity of heat shock during grain filling on dry matter and protein accumulation, technological quality and protein composition in bread and durum wheat. Funct. Plant Biol..

[57] Guo L., Wang Q., Chen H., Wu D., Dai C., Chen Y., Ma Y., Wang Z., Li H., Cao X. (2022). Moderate addition of B-type starch granules improves the rheological properties of wheat dough. Food Res. Int..

[58] Li H., Ma Y., Pan Y., Yu L., Tian R., Wu D., Xie Y., Wang Z., Chen X., Gao X. (2021). Starch other than gluten may make a dominant contribution to wheat dough mixing properties: A case study on two near-isogenic lines. LWT-Food Sci. Technol..

[59] Yu L., Ma Y., Zhao Y., Rehman A.U., Guo L., Liu Y., Yang Y., Wang Z., Cao X., Gao X. (2022). Interaction of B-type starch with gluten skeleton improves wheat dough mixing properties by stabilizing gluten micro-structure. Food Chem..

[60] Howden S.M., Soussana J.F., Tubiello F.N., Chhetri N., Dunlop M., Meinke H. (2007). Adapting agriculture to climate change. Proc. Natl. Acad. Sci. USA.

[61] Mamrutha H.M., Rinki K., Venkatesh K., Gopalareddy K., Khan H., Mishra C.N., Kumar S., Kumar Y., Singh G., Singh G.P. (2020). Impact of high night temperature stress on different growth stages of wheat. Plant Physiol. Rep..

[62] Hein N.T., Impa S.M., Dan W., Bheemanahalli R., Kumar R., Tiwari M., Prasad V.P.V., Tilley M., Wu X.R., Neilsen M. (2022). Grain micronutrient composition and yield components in field-grown wheat are negatively impacted by high nighttime temperature. Cereal Chem..

[63] Sekularac A., Torbica A., Živančev D., Tomić J., Knežević D. (2018). The influence of wheat genotype and environmental factors on gluten index and the possibility of its use as bread quality predictor. Genetika.

[64] R-Core-Team (2022). R: A Language and Environment for Statistical Computing.

