# Supplementary material for: Effect of High Nighttime Temperatures on Growth, Yield, and Quality of Two Wheat Cultivars During the Whole Growth Period
Source: Plants (Basel). 2024 Oct 31;13(21):3071. doi: 10.3390/plants13213071 (PMC11548653; doi:10.3390/plants13213071)
Supplement: Supplementary file 1 [file plants-13-03071-s001.zip › plants-3253644-Supplementary Material-revised-highlight.pdf]

Supplementary Materials

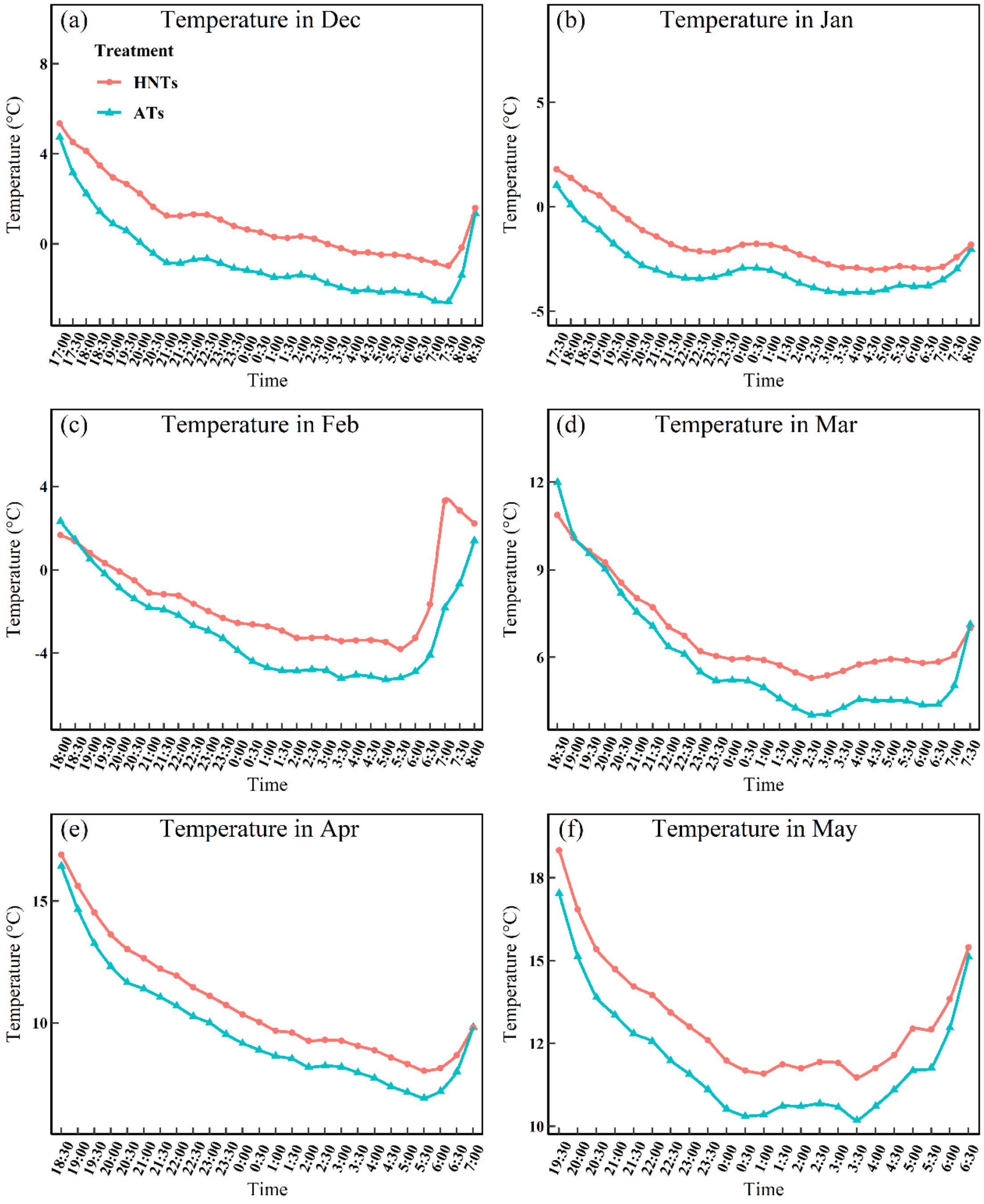

Figure S1. Differences in monthly mean nighttime temperatures between high nighttime temperatures (HNTs) and ambient temperatures (ATs).

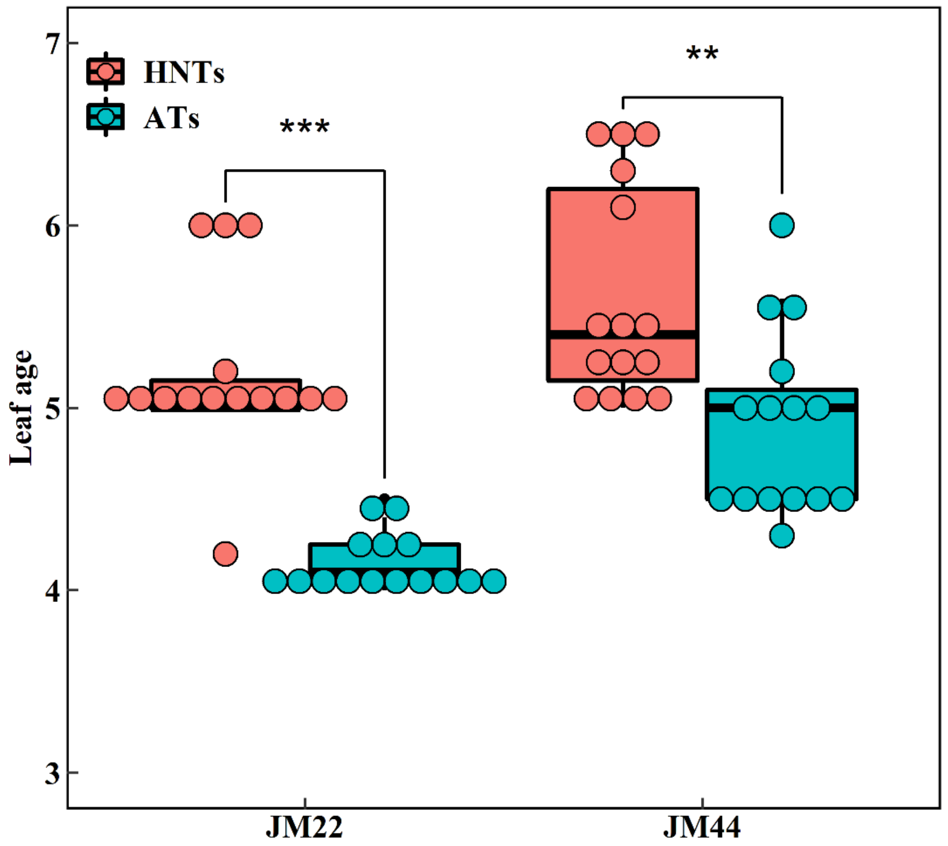

Figure S2. Leaf age of Jimai22 (JM22) and Jimai44 (JM44) at overwintering stage under the high nighttime temperatures (HNTs) and ambient temperatures (ATs). Analysis of variance (ANOVA) and the least significant difference (LSD) were used to test the significance of differences. **, and *** indicate significant difference between HNTs and ATs at the 0.01, and 0.001 levels, respectively.

Table S1. Effect of high nighttime temperatures on yield indices at harvest of Jimai22 (JM22) and Jimai44 (JM44).

Cultivar	Treatment	number of fertile spikelets (NFS)	number of sterile spikelets (NSS)	harvest index (HI)(%)
JM22	HNTs	15.3±0.6 ab	3.6±0.3 b	34.2 b
	ATs	16.9±0.6 a	2.0±0.3 c	41.5 a
JM44	HNTs	13.3±0.6 c	4.7±0.4 a	29.5 c
	ATs	15.2±0.6 b	3.6±0.2 b	30.7 c

HNTs, high nighttime temperatures; ATs, ambient temperatures. Analysis of variance (ANOVA) and the least significant difference (LSD) were used to test the significance of differences. Different lower-case letters in the same column show significant differences ($P < 0.05$) within cultivars (or treatments).
